# Neonatal Problems and Infancy Growth of Term SGA Infants: Does “SGA” Definition Need to Be Re-evaluated?

**DOI:** 10.3389/fped.2021.660111

**Published:** 2021-11-12

**Authors:** Saygin Abali, Serdar Beken, Eda Albayrak, Aysegul Inamlik, Burcu Bulum, Ezgi Bulbul, Gulten Zeynep Eksi, Zeynep Alize Ay, Melis Karabay, Didem Kaya, Muge Halici, Serap Semiz, Ayse Korkmaz

**Affiliations:** ^1^Department of Pediatrics, Pediatric Endocrinology, School of Medicine, Acibadem Mehmet Ali Aydinlar University, Istanbul, Turkey; ^2^Department of Pediatrics, Neonatology, School of Medicine, Acibadem Mehmet Ali Aydinlar University, Istanbul, Turkey; ^3^Department of Pediatrics, School of Medicine, Acibadem Mehmet Ali Aydinlar University, Istanbul, Turkey; ^4^School of Medicine, Acibadem Mehmet Ali Aydinlar University, Istanbul, Turkey

**Keywords:** small for gestational age, hypoglycemia, short stature, overweight, obesity

## Abstract

**Introduction:** The exact definition of small-for-gestational-age (SGA) infant is still controversial among clinicians. In this study, we aimed to understand which definition is better in terms of establishing both early postnatal problems and growth. In this way, we compared early neonatal problems and infancy growth of term infants with birth weight (BW) < -2 SDS and with BW between 10th percentile (−1.28 SDS) and −2 SDS.

**Methods:** A single center retrospective cohort study was conducted. Preterm infants, multiple gestations and newborns with any congenital anomalies were excluded from the study. Study group was defined as Group 1 (*n* = 37), infants BW < −2.00 SDS; Group 2 (*n* = 129), between −1.28 and −2.00 SDS; and Group 3 (*n* = 137), randomly selected newborns with optimal-for-gestational-age (BW between −0.67 and +0.67 SDS) as a control group.

**Results:** The incidence of severe hypoglycemia was highest in Group 1 (%10.8) and Group 2 and 3 had similar rates of severe hypoglycemia (0.8 and 0.7%, respectively). The incidence of polycythemia was 5.4% in Group 1 and was significantly higher than Group 3 (0.0%) while it was 2.3% in Group 2. Short stature (length < −2 SDS) ratio at the age of 1 and 2 years were similar in each group. Overweight/obesity ratio at the age of 1 were 9.5, 20.8 and 16.7% in each group, respectively (*p* = 0.509).

**Conclusion:** This study was planned as a pilot study to determine potential differences in the problems of hypoglycemia, polycythemia, and growth according to the differences in definition. Short term disturbances such as hypoglycemia and polycythemia are found to be higher in infants with a BW SDS below −2. From this point of view, of course, it will not be possible to change the routine applications immediately, however this study will be an initiative for discussions by making long-term studies.

## Introduction

Small-for-gestational-age (SGA) infants have several consequences. These infants have not only higher rates of morbidity and mortality in the neonatal period but also have higher risk of health problems in later life including short stature, puberty disorders, metabolic syndrome, and neurocognitive dysfunction (1, 2). Hence, it is imperative to define SGA infants in the neonatal period and initiate regular follow-up to achieve better health and growth outcome.

Despite causing significant health problems, the exact definition of SGA is still controversial among clinicians. The majority of pediatricians and neonatologists prefer to use World Health Organization's (WHO) recommendation (3), defining SGA as <10th percentile (−1.28 standard deviation score, SDS) of birth weight (BW) for gestational age whereas pediatric endocrinologists use International Societies of Pediatric Endocrinology and Growth Hormone Research Society recommendations (4), defining as weight and/or length at birth <2 SD from the mean (5). The discrepancy between neonatologists and endocrinologists may cause a dilemma during the follow-up period.

In this study, we aimed to understand which definition is better in terms of establishing both early postnatal problems and growth. In this way, we compared early neonatal problems and infancy growth of term infants with BW < −2 SDS and with BW between −1.28 and −2 SDS.

## Patients and Methods

A single center retrospective cohort study was conducted between January 2014 and December 2019 in Acibadem University, Atakent Hospital, Istanbul. During the study period, only inborn term infants were included in the study. Newborns who have any congenital anomalies, multiple gestations, and preterm infants (gestational age below 37^0/7^ week) were excluded from the study to avoid their confounding effects for neonatal problems and postnatal growth. BW SDS of all infants were calculated according to Kurtoglu et al. (6) national newborn references by Childmetrics (7). Infants are grouped according to their BW SDS. Groups were as follows:

*Group 1* (*n* = 37), infants BW < −2.00 SDS; *Group 2* (*n* = 129), BW between −2 SDS and −1.28 SDS (10th percentile); and *Group 3* (*n* = 137), randomly selected newborns with optimal-for-gestational-age (OGA, BW for gestational age between 25 and 75 percentile (between −0.67 and +0.67 SDS) as a control group.

**Figure 1:**
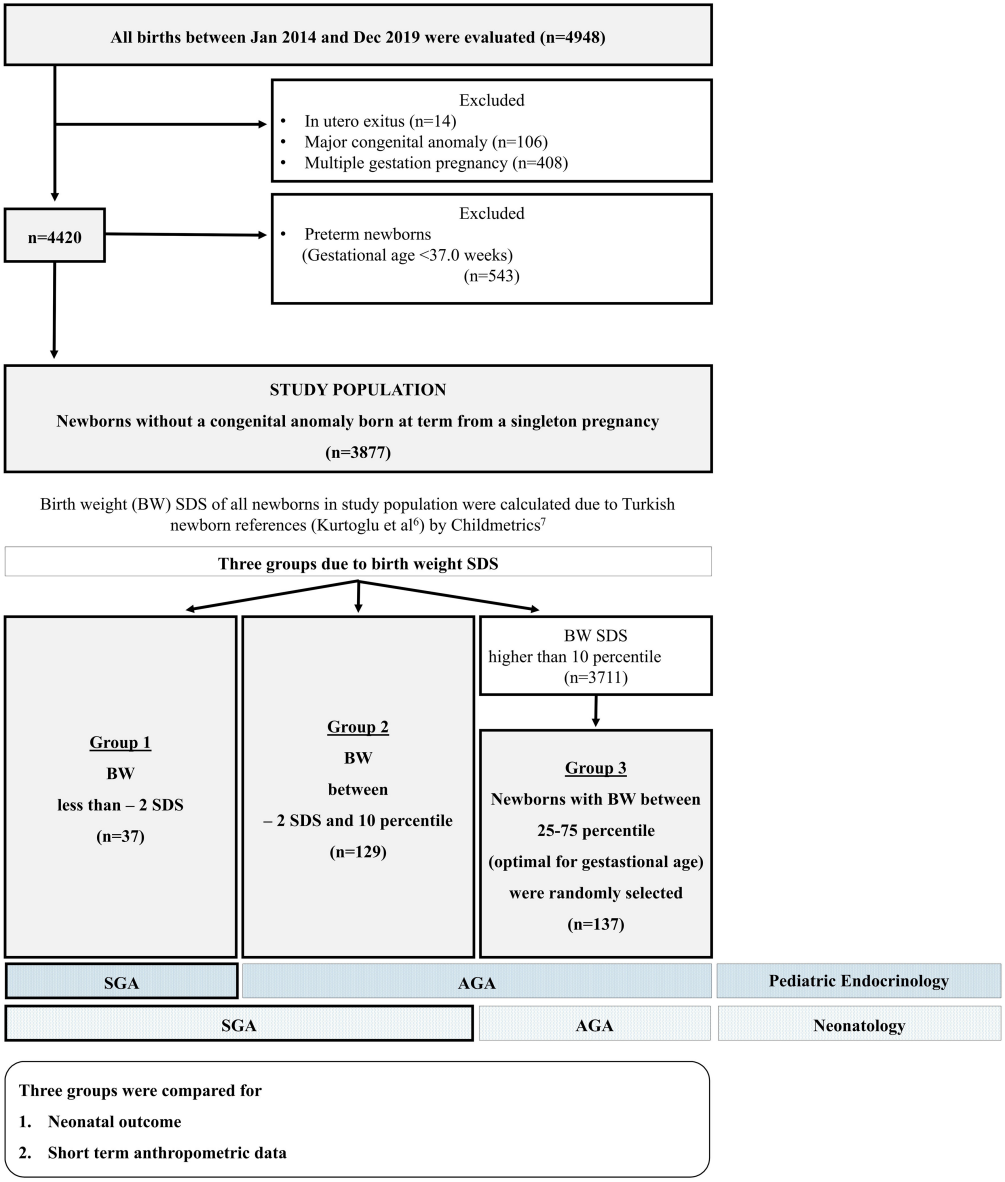
Sample selection and study design.

The method that we used in population selection was including all infants below −1.28 SDS (10th percentile) born in our hospital as study groups (Group 1 and 2) and, inborn OGA matches (gender, gestational age, and birth date appropriate) as control group. In the selection of the control group, OGA was preferred instead of AGA (10–90% percentile) in order to find the “ideal BW” according to the gestational age. Selection of the patients and study design was given in Figure 1.

Demographic data and birth anthropometry [gestational age, gender, BW, crown-heel length at birth, occipitofrontal circumference (OFC)] were recorded from electronic medical files. Clinical history, capillary blood glucose (described below) and also hemoglobin (Hb) levels at the postnatal 6th hour were recorded for early neonatal problems including hypoglycemia and polycythemia. Initial birth hospitalization data was also obtained. Anthropometric measurements (crown-heel length and body weight) at the age of 1 and 2 years were recorded for infancy growth follow-up.

### Hypoglycemia

According to our institutional protocol, in newborn infants without a risk for hypoglycemia, capillary blood glucose concentration was measured at least once at the 6th hour of life. Monitoring was not continued if the first glucose level was normal and if there were no clinical concerns about feeding. On the other hand, newborns with risk of hypoglycemia (such as BW below 10th and above 90th percentile, preterm infants and infants of diabetic mother) were closely monitored for their blood glucose levels by measuring at the postnatal 1st hour and before every feeding in every 3–4 h and then in the following day. Measurements were discontinued if three consecutive blood glucose level were within normal limits.

In this study, measured lowest capillary blood glucose level was taken to the statistical analysis.

For the definition and management of hypoglycemia, Turkish Neonatal Society (TNS) hypoglycemia guideline (8) [similar to the American Academy of Pediatrics 2011 (9)] was used in newborns. Plasma glucose concentrations <25 mg/dL within the first 4 h after birth and/or plasma glucose concentrations of <35 mg/dL between 4 and 24 h of age and/or newborns with abnormal clinical findings despite adequate feeding or intravenous (IV) glucose were defined as severe hypoglycemia.

### Polycythemia

According to hospital policy, a complete blood count is routinely performed at the 6th hour for all inborn babies, regardless of the risks. Polycythemia of the newborn was defined as venous hematocrit over 65%. As a risk factor for polycythemia delayed cord clamping or milking is not performed in term newborns in our institution.

### Hypothermia

Neonatal hypothermia is defined as an axillary temperature less than 36.5°C. Axillary temperature was measured in all newborns in their routine examination in nursery care room after birth.

### Hypoxic-Ischemic Encephalopathy

Hypoxic-ischemic encephalopathy diagnosis was based on a combination of medical history, having resuscitation after delivery, abnormal neurological exam, and abnormal cord blood gas results (10). All newborns were evaluated for HIE in delivery and cord blood sample was drawn from those with risk.

### Hospitalization

According to hospital policy, all newborn infants whose BW were below 2,000 g were followed in neonatal intensive care unit (NICU) at least for 24 h regardless of the clinical condition.

Addition to low BW indication, reasons for initial birth hospitalization were recorded (severe hypoglycemia, feeding problems, and respiratory distress).

In our institution, all infants were fed exclusively with breast milk if there is no problem such as hypoglycemia, respiratory distress.

### Anthropometric Evaluation

Anthropometric measurements of all patients were performed with standard methods by infantometer (Seca Mod. 207, Germany) (sensitive 0.1 cm). Weight was measured using a electronic scale (Seca GmBh&Co., kg, Hamburg, Germany) (sensitive to 5 g). Standard deviation score (SDS) of all measurements according to Turkish standards were calculated (7, 11). Ponderal index (PI) at birth of each patient was calculated as 1,000× [(BW (g)/BL (cm^3^)] and grouped as <10th and ≥10th percentile (12). Weight/ideal weight for length (W/L) at 1 and 2 years of age were calculated as 100× [patient's weight (kg)/ideal weight (50th percentile) at length age of the patient (kg)]. W/L below 90% was defined as malnutrition; between 110 and 120% as overweight and >120% as obesity (13).

Groups were compared for neonatal outcomes and infancy growth parameters.

### Statistical Analysis

Statistical Package for the Social Sciences program (SPSS version 16.0, Inc. Chicago, Illinois, USA) was used to analyze the data. While evaluating the study values, descriptive statistical methods (mean, standard deviation, median, frequency, and percentage) were used. The suitability of normal distribution of the quantitative data was tested by Shapiro–Wilk test and graphical analysis. Student's *t*-test was used for comparisons of normally distributed quantitative variables between two groups, and Mann–Whitney *U*-test was used for comparisons of not normally distributed quantitative variables between two groups. Kruskal–Wallis test was used to compare not normally distributed quantitative variables for three groups or more, and for their pairwise comparisons Bonferroni-Dunn test was used. Pearson Chi-Square test and Fisher–Freeman–Halton test were used for comparison of qualitative data. The relationship between the data were analyzed using Pearson correlation (correlation coefficient: *r*). Statistical significance was accepted as *p* < 0.05.

A *post-hoc* power analysis was applied based on plasma glucose levels, and the power of the study was calculated as 99.6% with an effect size of 0.875 and an alpha level of 5% (G^*^Power 3.1.9.7 for Windows XP).

The study was approved by the Acibadem Mehmet Ali Aydinlar University Ethics Committee (ATADEK 2019-1/36). The study was retrospective and did not involve interventions, thus informed consent from the parents and patients was not obtained.

## Results

Demographic characteristics and birth anthropometry of the study population were given in Table 1. BL SDS at birth was below −2 in only 1 newborn in Group 1. In all other newborns BL SDS were above −2. Ratio of the patients PI <10th percentile in groups were 37.8, 12.4, and 0.0%, respectively (*p* < 0.001; Table 1).

**Table 1:** Demographic characteristics and birth anthropometry of the study population.

	**Group 1**	**Group 2**	**Group 3**	***p* ^**1**^**	***p* ^**2**^**	***p* ^**3**^**
	***n =* 37**	***n =* 129**	***n =* 137**			
**GW** (wk), mean ± SD	39.0 ± 1.0	38.8 ± 1.0	38.9 ± 1.1	0.302	0.157	0.946
**Girls**, *n* (%)	14 (37.8)	68 (52.7)	71 (51.8)	0.192	0.885	0.223
**VD**, *n* (%)	4 (10.8)	30 (23.3)	32 (23.4)	0.098	0.984	0.095
**BW** (g), mean ± SD	2,439.7 ± 189.7	2,647.3 ± 183.5	3,237.5 ± 164.8	**<0.001**	**<0.001**	**<0.001**
**BW SDS**, mean ± SD (median)	−2.33 ± 0.37 (−2.21)	−1.53 ± 0.20 (−1.49)	0.04 ± 0.23 (0.04)	**0.001**	**0.001**	**0.001**
**BL** (cm), mean ± SD	47.9 ± 1.1	48.3 ± 1.6	50.4 ± 1.3	**0.149**	**<0.001**	**<0.001**
**BL SDS**, mean ± SD (median)	−0.96 ± 0.48 (−0.87)	−0.64 ± 0.69 (−0.80)	0.40 ± 0.65 (0.47)	**0.017**	**0.001**	**0.001**
**Head C** (cm), mean ± SD	32.7 ± 1.1	33.1 ± 1.1	34.6 ± 1.0	**0.070**	**<0.001**	**<0.001**
**Head C SDS**, mean ± SD (median)	−1.49 ± 0.72 (−1.32)	−1.10 ± 0.74 (−1.05)	0.03 ± 0.82 (−0.08)	**0.006**	**0.001**	**0.001**
**Chest C** (cm), mean ± SD (median)	30.1 ± 1.7 (30.0)	30.9 ± 1.3 (31.0)	33.0 ± 1.2 (33.0)	**0.004**	**0.001**	**0.001**
**Ponderal index**, mean ± SD (median)	22.2 ± 1.8 (22.0)	23.6 ± 1.9 (23.8)	25.3 ± 1.8 (25.0)	**<0.001**	**<0.001**	**<0.001**
**PI < 10th p**, *n* (%)	14 (37.8)	16 (12.4)	0 (0.0)	**0.001**	**<0.001**	**<0.001**

*GW, gestational week; VD, vaginal delivery; BW, birth weight; SDS, Standard deviation score; BL birth length (crown-heel length); C, circumference; PI, Ponderal index; 10p, 10 percentile*.

*p^1^ is between Group 1 and 2; p^2^ is between Group 2 and 3; p^3^ is between Group 1 and 3*.

*Bold values used for significant p values*.

### Hypoglycemia

**Figure 2:**
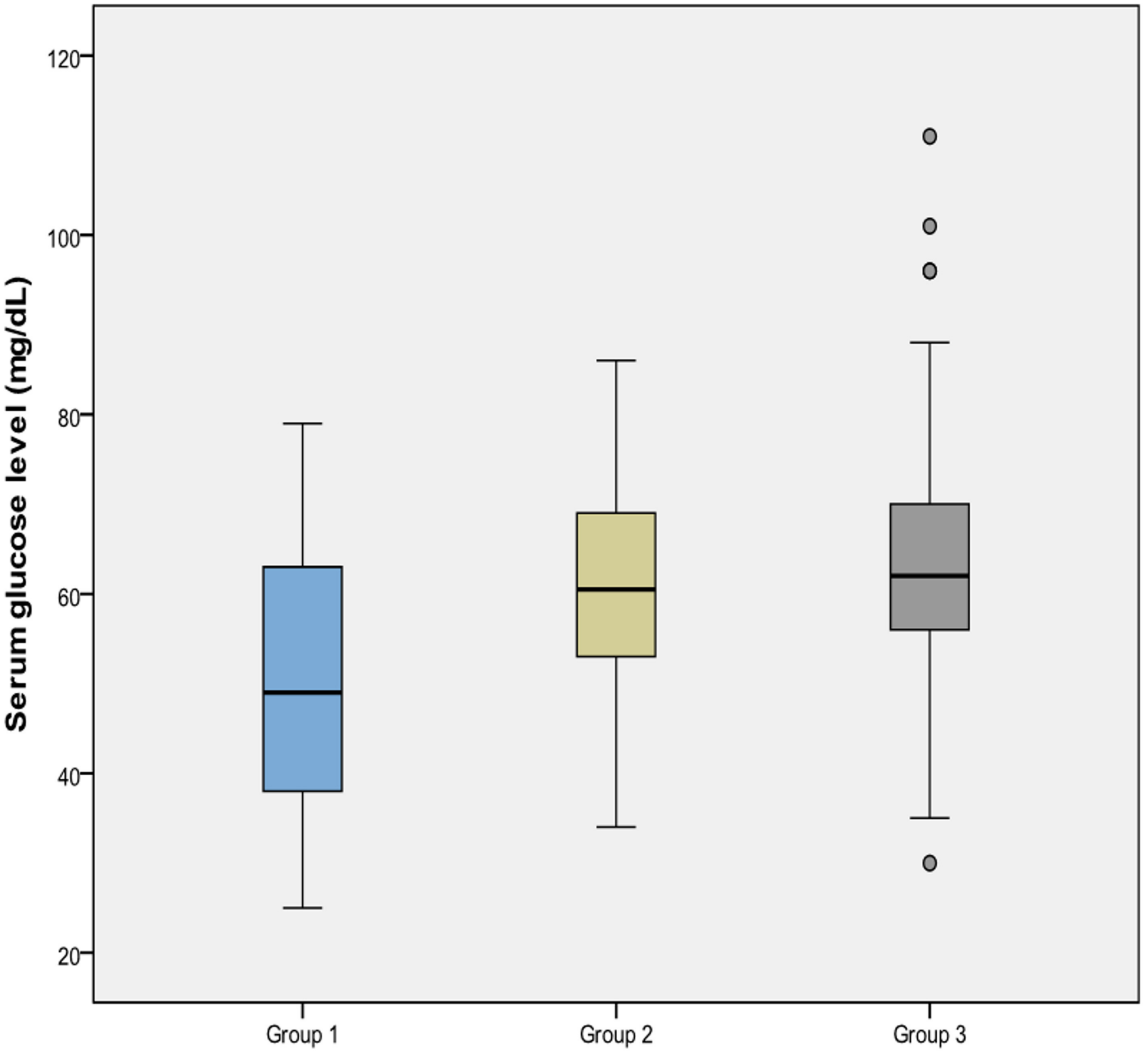
Comparison of serum glucose levels of the groups.

Measured lowest capillary blood glucose was presented in Table 2. The mean glucose concentration of Group 1 was lower than that of both Group 2 and Group 3 (*p* = 0.001), however Group 2 and 3 were similar (*p* = 0.264) for this value (Figure 2). The incidence of severe hypoglycemia was also highest in Group 1 (%10.8) (*p* = 0.008 and 0.009, respectively), and Group 2 and 3 had similar severe hypoglycemia ratio (0.8 and 0.7%, respectively, *p* = 0.956). PI at birth was negatively correlated with glucose concentration (*r* = −0.283, *p* < 0.001).

**Table 2:** Clinical characteristics and incidences of neonatal morbidities of the study population.

	**Group 1**	**Group 2**	**Group 3**	***p* ^**1**^**	***p* ^**2**^**	***p* ^**3**^**
	***n* = 37**	***n* = 129**	***n* = 137**			
**Blood glucose** (mmol/L), mean ± SD (median)	2.7 ± 0.8 (2.8)	3.4 ± 0.7 (3.4)	3.5 ± 0.7 (3.4)	**0.001**	0.264	**0.001**
**Postnatal 6th hour hemoglobin** g/dl, mean ± SD (median)	20.1 ± 1.9 (20.1)	18.4 ± 1.8 (18.4)	17.5 ± 1.7 (17.5)	**0.001**	**0.001**	**0.001**
**Hospitalization**, *n* (%)	9 (24.3)	9 (7.0)	4 (2.9)	**0.003**	0.125	**0.001**
Severe hypoglycemia, *n* (%)	4 (10.8)	1 (0.8)	1 (0.7)	**0.009**	0.956	**0.008**
Polycythemia, *n* (%)	2 (5.4)	3 (2.3)	0 (0.0)	0.309	0.113	**0.044**
Feeding problems, *n* (%)	1 (2.7)	1 (0.8)	1 (0.7)	0.397	0.966	0.318
Respiratory distress, *n* (%)	0 (0.0)	4 (3.1)	2 (1.5)	0.576	0.435	0.460
BW < 2,000 g	2 (5.4)	0 (0.0)	0 (0.0)	**0.049**	1.000	**0.044**

*BW, birth weight; SD, standard deviation*.

*p^1^ is between Group 1 and 2; p^2^ is between Group 2 and 3; p^3^ is between Group 1 and 3*.

*Bold values used for significant p values*.

### Polycythemia

Hb concentrations at 6th hour was presented in Table 2. Mean Hb concentrations of each group were different significantly from each other and it was highest in Group 1. The incidence of polycythemia was 5.4% in Group 1 and was significantly higher than Group 3 (0.0%) (*p* = 0.044) while it was 2.3% in Group 2 (*p* = 0.113).

PI at birth was positively correlated with Hb concentration (*r* = 0.166, *p* = 0.004).

### Other Neonatal Outcomes

Initial birth hospitalization ratio of the groups is given in Table 2. None of the patients in each group had perinatal hypothermia and hypoxic-ischemic encephalopathy.

### Anthropometric Follow-Up Data

At the age of 1 year, only one infant had body length below 3rd percentile in Group 2. All patients had height catch-up growth in Group 1. Short stature (length < 3rd percentile) ratio at the age of 1 year were similar in each group (*p* = 0.490). The percent of having W/L below 90.0% in each group were 19.0, 11.3, and 0.0%, respectively (*p* = 0.010). Overweight/obesity (W/L > 110%) ratio were 9.5, 20.8, and 16.7% in each group, respectively (*p* = 0.509).

At the age of 2 years, short stature (length < 3rd percentile) ratio were similar in each group (*p* = 0.830). The percent of having W/L below 90.0% in each group were 42.9, 30.8, and 10.8%, respectively (*p* = 0.030). Overweight/obesity (W/L > 110%) ratio were 7.1, 3.8, and 24.3% in each group, respectively (*p* = 0.051).

**Figure 3:**
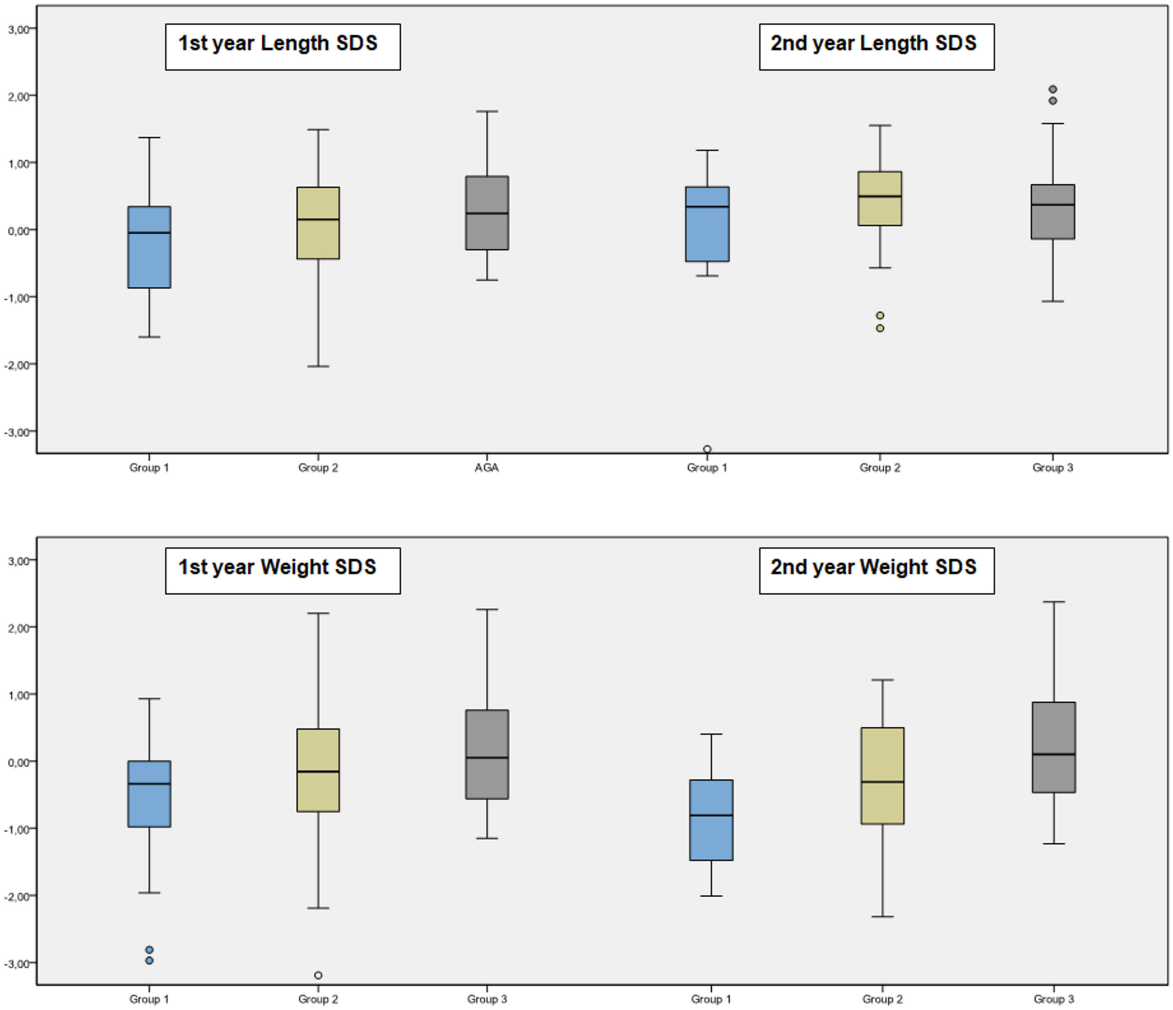
Comparison of weight and length SDs of the groups.

Anthropometric follow-up data was presented in Table 3 and Figure 3.

**Table 3:** Anthropometric follow-up data.

	**Group 1**	**Group 2**	**Group 3**	***p* ^**1**^**	***p* ^**2**^**	***p* ^**3**^**
**1st year anthropometry**	***n* = 22**	***n* = 53**	***n* = 54**			
**Age** (years)	1.0 ± 0.1	1.0 ± 0.2	1.0 ± 0.1	ns		
**Length SDS**, mean ± SD (median)	−0.29 ± 0.79 (−0.06)	0.07 ± 0.86 (0.15)	0.28 ± 0.67 (0.25)	0.082	0.257	**0.015**
**Weight SDS**, mean ± SD (median)	−0.67 ± 0.99 (−0.51)	−0.13 ± 1.06 (−0.15)	0.13 ± 0.86 (0.05)	0.064	0.274	**0.005**
**W/L %**, mean ± SD (median)	96.5 ± 9.1 (99.2)	101.5 ± 11.3 (100.4)	103.0 ± 9.0 (101.4)	0.132	0.400	**0.016**
**Length < 3rd**, *n* (%)	1 (4.5)	0 (0.0)	0 (0.0)	0.490		
**W/L <90%**, *n* (%)	4 (18.2)	6 (11.3)	0 (0.0)	0.381	**0.011**	**0.001**
**W/L>110%**, *n* (%)	2 (9.1)	11 (20.7)	9 (16.7)	0.252	0.588	0.432
**2nd year anthropometry**	***n*** **= 14**	***n =*** **27**	***n =*** **41**			
**Age** (years)	2.0 ± 0.3	2.0 ± 0.2	2.1 ± 0.4	ns		
**Length SDS**, mean ± SD (median)	−0.25 ± 1.48 (0.10)	0.40 ± 0.77 (0.49)	0.37 ± 0.75 (0.38)	0.200	0.529	0.322
**Weight SDS**, mean ± SD (median)	−1.07 ± 1.12 (−0.82)	−0.39 ± 1.18 (−0.31)	0.18 ± 0.93 (0.10)	0.064	0.094	**0.001**
**W/L %**, mean ± SD (median)	93.5 ± 10.9 (91.2)	95.8 ± 7.8 (94.9)	100.9 ± 9.4 (98.6)	0.171	**0.042**	**0.010**
**Length < 3rd**, *n* (%)	1 (7.1)	0 (0.0)	0 (0.0)	0.830		
**W/L < 90%**, *n* (%)	4 (28.6)	6 (22.2)	0 (0.0)	0.445	**0.047**	**0.010**
**W/L > 110%**, *n* (%)	2 (14.3)	11 (40.7)	9 (21.9)	0.648	**0.029**	0.168

*W/L, weight for length; p^1^ is between Group 1 and 2; p^2^ is between Group 2 and 3; p^3^ is between Group 1 and 3*.

*Bold values used for significant p values*.

There was no correlation between PI at birth and anthropometric measures at the ages of 1 and 2 years.

## Discussion

SGA is defined as BW below the 10th percentile usually by a pediatrician or neonatologist. However, the infant is not “small” enough for the pediatric endocrinologist who accept −2 SDS as the cutoff. The definition of SGA does not match perfectly. Manuscripts in the literature also reflect this situation; while the majority of the publications written about SGA infants in pediatric journals have used a BW below the 10th percentile as definition whereas endocrinology journals have used the definition a BW below −2 SDS (5). Although the definition is controversial, it is important to determine these vulnerable population of infants and initiate close follow-up. Term SGA or “small” infants do not have the complications related to organ system immaturity that premature infants of similar size have. However, they are at risk of perinatal asphyxia, meconium aspiration, hypoglycemia, polycythemia, and hypothermia (14).

Hypoglycemia is one of the most common problems seen in neonates in the nursery and NICU. Even 10% of healthy term infants have hypoglycemia risk in the first 24–48 h after birth; SGA, large-for-gestational-age, late preterm infants, and infants of diabetic mothers have an additional risk (14). Inadequate glycogen and reduced fat sources for gluconeogenesis and also high insulin levels in some cases are responsible for hypoglycemia in SGA infants (15). The estimated incidence of hypoglycemia in SGA infants is around 10–50% (15, 16). In our study, the incidence of severe hypoglycemia was found to be ~11% in newborns with a BW below −2 SDS, and only 1.0% in infants with a BW SDS between −1.28 and −2. Babies with additional risks were excluded from the study, therefore severe hypoglycemia is thought to be lower in our study. In a recent study in which the definition of SGA was below the 3rd percentile (~ <-2 SDS), it was reported that the rate of SGA was 7.5% (9/119), and none of them had severe hypoglycemia (16).

Similar to our study, Mejri et al. (15) compared term newborn infants with a BW between 5th and 10th percentile (−1.28 and −1.64 SDS) and <5th percentile (<-1.64 SDS). However, they found no difference between the incidences of hypoglycemia and severe hypoglycemia (need for IV glucose) between those two groups. In our study, severe hypoglycemia was more common among infants with a BW below −2 SDS. In line with the AAP recommendations (17), as much as possible blood glucose regulation was tried to be provided with enteral nutrition. In infants with a BW below −2 SDS, despite proper control, IV glucose requirement as a result of severe hypoglycemia was higher, indicating that real-risk babies are in this group.

PI was used for the nutritional status of the newborn and neonatal malnutrition was defined as the PI < 10th percentile. Addition to being SGA, low PI is also associated with poor outcome (18). We have also demonstrated PI and glucose association.

Children born SGA represent a heterogeneous group at risk for short stature, obesity, and metabolic complications (19–22). Up to 90% of the SGA infants experience an accelerated growth during the first year of life that results in a height above −2 SDS. Most of the catch-up growth occurs during the first year and is nearly completed by the age of 2 years (4, 23, 24). It was reported that children born premature, SGA and with severe growth retardation, especially with reduced birth length, are less likely to reach a normal height (25). In our cohort, there was only one newborn with reduced birth length, and we have excluded preterm infants. Therefore, in our study, almost 95% of the infants born SGA completed their catch-up growth in height in the first year.

This catch-up growth may have long-term benefits on achieving a normal height (4, 26, 27). However, it might also result in metabolic disturbances later in life (26–29). Birth cohort studies have shown that reduced BW is usually followed by rapid infant weight gain (28,30). In our study, at second year, ~1/3 of the SGA infants were overweight or obese while only 1/5 of those born OGA were overweight or obese. This ratio was significantly higher (40%) in SGA infants with a BW above −2 SDS.

Different definitions for SGA at different stages from medical education to clinical practice is a dilemma. This confusion should be resolved. We think that while the statistically logical definition of pathological deviation is being <2 SD from the mean, it would be necessary to discuss the traditional definition. At least, reducing the frequency and duration of blood glucose monitoring and eliminating the need for screening for polycythemia are some of the situations that can be beneficial in the neonatal period. In long term follow-up, when a patient with short stature is referred to pediatric endocrinology, it is important to know whether the patient was born SGA or not in terms of both investigating the etiology of short stature and growth hormone treatment.

### Study Limitations

The limitation of this study was being retrospective. Hypoglycemic attacks, which were considered severe and require NICU hospitalization, were included in the study; while hypoglycemic events, which were followed in the nursery with exclusive breastfeeding in line with the TNS and AAP guidelines were not evaluated. It was thought that it would be appropriate to evaluate and compare hypoglycemic episodes which did not require intravenous glucose in newborns who are between −1.28 and −2.00 SDS with prospective clinical observational studies.

Since it is a retrospective study, maternal data could not be obtained under optimal conditions. For this reason, children of diabetic mothers could not be excluded.

And also, since our hospital started accepting patients since 2014, the number of the patients with a BW below −2 SDS and our follow up data were limited. Long term follow-up data for neurodevelopmental milestones, the components of metabolic syndrome and pubertal timing also were missing.

## Conclusion

This study was planned as a pilot study to determine potential differences in the problems of hypoglycemia, polycythemia, and growth according to the differences in definition. Short term disturbances such as hypoglycemia and polycythemia are found to be higher in infants with a BW SDS below −2. From this point of view, of course, it will not be possible to change the routine applications immediately, however, this study will be an initiative for discussions by making long-term studies.

## Data Availability Statement

The raw data supporting the conclusions of this article will be made available by the authors, without undue reservation.

## Ethics Statement

The studies involving human participants were reviewed and approved by Acibadem Mehmet Ali Aydinlar University Ethics Committee (ATADEK 2019-1/36). Written informed consent to participate in this study was provided by the participants' legal guardian/next of kin.

## Author Contributions

SB, EA, and AK: medical practices. SA, SB, and BB: concept. SA, SB, BB, SS, and AK: design. SA, SB, EA, AI, EB, GE, ZA, MK, DK, and MH: data collection or processing. SA and SB: analysis or interpretation. SA, SB, SS, and AK: literature search and writing. All authors discussed the results, commented on the manuscript, and have accepted responsibility for this submitted manuscript and approved submission.

## Conflict of Interest

The authors declare that the research was conducted in the absence of any commercial or financial relationships that could be construed as a potential conflict of interest.

## Publisher's Note

All claims expressed in this article are solely those of the authors and do not necessarily represent those of their affiliated organizations, or those of the publisher, the editors and the reviewers. Any product that may be evaluated in this article, or claim that may be made by its manufacturer, is not guaranteed or endorsed by the publisher.
